# Isoginkgetin protects chondrocytes and inhibits osteoarthritis through NF-κB and P21 signaling pathway

**DOI:** 10.1186/s10020-025-01302-6

**Published:** 2025-06-22

**Authors:** Mengdai Xu, Xi Chen, Shasha Du, Huanhuan Xu, Changyu Liu

**Affiliations:** 1 Department of Clinical Nutrition, Traditional Chinese and Western Medicine Hospital of Wuhan, Tongji Medical College, Huazhong University of Science and Technology, Wuhan, Hubei 430022 People’s Republic of China; 2 Department of Pharmacy, Traditional Chinese and Western Medicine Hospital of Wuhan, Tongji Medical College, Huazhong University of Science and Technology, Wuhan, Hubei 430022 People’s Republic of China; 3 Department of Obstetrics and Gynecology, Wuhan Children’s Hospital, Tongji Medical College, Huazhong University of Science and Technology, 100 Xianggang Road, Wuhan, 430000 China; 4 Department of Orthopedics, Tongji Hospital, Tongji Medical College, Huazhong University of Science and Technology, 1095 Jiefang Ave, Wuhan, Hubei 430030 China; 5 Department of Clinical Nutrition, Wuhan No.1 Hospital, Wuhan, Hubei 430022 People’s Republic of China; 6 Department of Pharmacy, Wuhan No.1 Hospital, Wuhan, Hubei 430022 People’s Republic of China; 7 Hospital Peoples of Daye City, the Second Affiliated of Hubei Polytechnic University, No.26 Donggang Road, Luojiaqiao Street, Daye City, 435100 Hubei China

**Keywords:** Isoginkgetin, Osteoarthritis, Inflammation, Pain, IL-1β

## Abstract

**Objective:**

Osteoarthritis (OA) is the most prevalent chronic articular disease in adults. The degree of cartilage degradation and matrix depletion in OA have been substantially connected with chondrocyte inflammatory response. Consequently, pharmacological anti-inflammatory agents provide OA patients a new therapeutic option. Isoginkgetin (IGK), a bioactive bioflavonoid derived from the medicinal herb Ginkgo Biloba, defends against obesity-induced heart diastolic dysfunction and harmful remodeling. Whether IGK has a regulatory effect on OA remains unknown. This study investigated whether IGK could attenuate the progression of OA both in vivo and in vitro.

**Methods:**

Cell Counting Kit-8 (CCK8) was used to measure the vitality of chondrocytes. Mediators of inflammation, anabolism and catabolism were tested by Western blot and RT-PCR. Safranin-O staining, Hematoxylin–Eosin (H&E) staining, immunofluorescence, and Osteoarthritis Research Society International (OARSI) standards were used to assess the severity of OA and the degradation of articular cartilage. The phenotype of cartilage, NF-κB and P21 signaling pathway were measured by Western Blot. The mRNA sequencing was selected to find the differentially expressed genes and potential pathway. Pain of mice was measured by Von Frey hair mechanosensitivity. The senescence level of chondrocyte was SA-β-Gal staining.

**Results:**

IGK inhibited catabolism and promoted anabolism after stimulating by IL-1β in vitro. Following destabilization of the medial meniscus (DMM) surgery, administration of IGK significantly reduced OARSI scores and attenuated AGGRECAN and COLLAGEN2 loss, overexpression of MMP3 and articular cartilage deterioration. IGK relieved pain of mice after DMM. Besides, PI3K/AKT/NF-κB, P53, Autophagy, Ferroptosis pathway and reactive oxygen species (ROS), senescence of cartilage were changed after IGK treatment.

**Conclusion:**

IGK protects articular cartilage and reduces the progression of OA in a mouse model and shows promise as a potential therapeutic strategy for OA.

## Introduction

Osteoarthritis (OA) is a common degenerative joint disease characterized by pain, swelling, restricted mobility, and deformity (Glyn-Jones et al. 2015). OA has become one of the most common chronic conditions causing physical limitations and a lower quality of life in the elderly population as a result of an increase in life expectancy (Li et al. 2018). Reducing knee-joint activity, giving anti-inflammatory and analgesic treatment are recommended for patients with early OA; joint replacement surgery is advised for end-stage patients to alleviate knee-joint pain and restore certain functions (Mandl 2019). OA has placed a heavy economic burden on society because of its high incidence, long treatment cycle, and side effects from pharmacological therapy (Palazzo et al. 2016). Therefore, finding appropriate medications or investigating the etiology of OA is a way to address this problem.

The cartilage is the most important part of the joint and has a direct impact on the onset and progression of OA (Pritzker et al. 2006). As the only cell type in articular cartilage, chondrocytes protect the integrity of the cartilage by regulating the production and breakdown of extracellular matrix (ECM) (Sandell and Aigner 2001). ECM deterioration is caused by increased catabolism and decreased anabolism in chondrocyte (Mobasheri et al. 2017). Although the pathophysiology of OA is unclear, chronic inflammation (Bosch 2021), reactive oxygen species (ROS) (Hui et al. 2016) and senescence-associated secretory phenotype (SASP) (Coryell et al. 2021; Freund et al. 2010) are thought to be main factors. Enhanced inflammatory response is highly correlated with activation of Mitogen-Activated Protein Kinase (MAPK) and Nuclear Factor B (NF-κB) pathways (Ostojic et al. 2021; Li et al. 2020; Wei et al. 2020; Wei et al. 2025).

It has been reported that a variety of flavonoids can inhibit cartilage inflammation and alleviate OA progression. Icariin exerts an inhibitory effect on chondrocyte ferroptosis and mitigates osteoarthritis via the up-regulation of the SLC7A11/GPX4 signaling pathway (Xiao et al. 2024). Luteolin inhibits IL-1β induced inflammation in rat chondrocytes by inhibiting phosphorylation of NF-κB (Fei et al. 2019). Isoginkgetin (IGK), as a natural flavonoid, was initially identified in the traditional Chinese medication Ginkgo (Briançon-Scheid et al. 1983). IGK attenuates monoamine neurotransmitter deficiency and depression-like behaviors through downregulating the p38/NF-κB inflammatory signaling pathway (Li et al. 2021), and protects against obesity-induced cardiomyopathy (Wu et al. 2022). Moreover, it has been demonstrated that IGK reduced the expression of inflammatory factors, such as NO synthase, COX-2, TNF-α, IL-6, and prostaglandin E2 (PGE2) in LPS-activated RAW 264.7 macrophage cells (Li et al. 2019a). Therefore, we hypothesize that IGK may reduce cartilage degradation through anti-inflammatory pathways. In this study, we investigated the function of IGK in OA in vitro and in vivo.

## Materials and methods

### Isolation and culture of mice chondrocytes

Chondrocytes were obtained from 6-day-old C57BL/6 mice. Cartilage was separated from the knee and ankle joints. Next, the cartilage was digested with 0.25% trypsin (Boster, China) at 37℃ for 30 min. After the trypsin was removed, the tissue was digested at 37℃ for 5 h using 0.25% type II collagenase (Sigma, USA). The cells were subsequently collected and grown in DMEM/F12 (Hyclone, USA) medium with 10% fetal bovine serum (FBS) at 37℃ in a humidified atmosphere of 5% CO_2_.

### IGK administration

IGK (Sinopharm Chemical Reagent Co., Ltd. China) was dissolved in dimethyl sulfoxide (DMSO) to a concentration of 20 mM and stored at −20 °C. In vitro, chondrocytes were treated with or without IL-1β (5 ng/ml) and different concentration of IGK. In vivo, 5 μl of a 20 μM solution of IGK and the same amount of PBS were injected into knee cavity.

### Measurement of cell viability

Cell Counting Kit—8 assay (CCK8, Boster, China) was used to evaluate the effects of IGK on chondrocyte viability. In 96-well plates, chondrocytes (8000 cells/well) were seeded, allowed to adhere for 24 h, and then treated as mentioned above.

### RNA extraction and quantitative RT-PCR

After interfering chondrocytes with IGK for 24 h, total RNA was isolated using the FastPure® Cell/Tissue Total RNA Isolation Kit V2 (Vazyme, China) following the manufacturer’s instructions. 1 μg RNA was reverse-transcribed into cDNA with the HiScript II cDNA Kit (Vazyme, China). The cDNA, SYBR Green Master mix (Vazyme, China), and primers were combined in the appropriate ratios and subjected to amplification and measurement using an RT-qPCR detection device (Biorad, USA). The relative mRNA levels were normalized for quantification against the levels of β-Actin. The primer sequences utilized in the experiment are presented in Table 1.

**Table 1:** Mouse primer sequences for RT-PCR

Gene		Primer sequence (5′–3′)
*β-Actin*	F	GGCTGTATTCCCCTCCATCG
	R	CCAGTTGGTAACAATGCCATGT
*Col2α1*	F	GGGAATGTCCTCTGCGATGAC
	R	GAAGGGGATCTCGGGGTTG
*Acan*	F	GTGGAGCCGTGTTTCCAAG
	R	AGATGCTGTTGACTCGAACCT
*Mmp3*	F	ACATGGAGACTTTGTCCCTTTTG
	R	TTGGCTGAGTGGTAGAGTCCC
*Mmp13*	F	CTTCTTCTTGTTGAGCTGGACTC
	R	CTGTGGAGGTCACTGTAGACT
*Cox2*	F	TTCAACACACTCTATCACTGGC
	R	AGAAGCGTTTGCGGTACTCAT
*iNOS*	F	GTTCTCAGCCCAACAATACAAGA
	R	GTGGACGGGTCGATGTCAC

### Western blotting analysis

The chondrocytes were treated with IL-1β and IGK for 24 h. After being washed with phosphate-buffered saline (PBS; Boster Bio Tech, China) three times, the chondrocytes were lysed using enhanced RIPA lysate containing 1% protease and phosphatase inhibitor (Boster, China). 10 μg of protein was separated using 10% SDS-PAGE gels and was subsequently transferred to a polyvinylidene difluoride membrane (Millipore, USA). Then, 5% bovine serum albumin (BSA, BioFroxx, Germany) was used to block the membrane for 1 h. The membrane was incubated with corresponding primary antibodies at 4 °C overnight and rinsed three times with tris-buffered saline containing 0.1% Tween 20 (GServicebio, China). The membrane was subsequently incubated with corresponding secondary antibodies (Boster, China) for 1.5 h. The protein bands were detected using the Image Lab System (Bio-Rad, USA).

### Animal procedure

Eleven-week-old male C57 mice were purchased from Beiente Biotechnology Co., Ltd. Surgical destabilization of the medial meniscus (DMM) was performed to create the OA model in the right knee joint under general anesthesia. In the sham-operated mice group, the joint was surgically exposed without performing ligament transection. Before the DMM procedure, the mice underwent a one-week adaptation period. The mice were randomly divided into three groups, with six mice per group (Sham group, DMM group and DMM + IGK injection group). Mice in the DMM + IGK group were administered 20 μM IGK. After the DMM surgery, based on previous studies,, 5 μL of IGK or PBS was injected into the knee joint cavity using a 33G needle (Hamilton, Bonaduz, GR, Switzerland). The injection was performed once a week, starting 1 week after the surgery and continuing for a total of 8 weeks (Tao et al. 2021). After 8 weeks, all mice were euthanized and their right knee joints were collected.

### Von Frey hair mechanosensitivity

Secondary allodynia was measured by Von Frey hair mechanosensitivity according to the experimental protocol (Deuis et al. 2017). The mice are first acclimated to the testing environment. Then, a series of Von Frey filaments (North Coast, USA) with varying force levels are applied to their right feet. The force of each filament gradually increases until the mouse displays a withdrawal response, such as flinching or paw lifting. The mechanical force required to elicit a paw withdrawal response in 50% of animals is determined using the “up-down” Von Frey method.

### Histological assessment

The harvested knee joint samples were immersed in a 4% paraformaldehyde solution (GServicebio, China) for 3 days. In the following stage, decalcification of the samples was carried out using a 10% EDTA decalcifying solution (Boster, China) for 2 weeks. The samples were then embedded in paraffin wax for further analysis. Different staining techniques such as Hematoxylin and eosin (HE), safranin O-Fast green (S.O.), and immunofluorescence were performed. The evaluation of articular cartilage degradation was done using the Osteoarthritis Research Association (OARSI) histopathological grading system.

### mRNA sequencing

RNA-seq were performed by Wuhan IGENEBOOK Biotechnology Co.,Ltd (http://www.igenebook.com). Briefly, whole-transcriptome sequencing was employed in primary chondrocytes after induced by IL-1β and IL-1β + IGK for 24 h. Three independent replicates were established for each experimental group. The integrity of all RNA samples was evaluated using a Qsep400 instrument. For the construction of RNA libraries with the MGIEasy mRNA Library Prep Kit, a total of 3 μg of RNA was utilized (Xu et al. 2019). The procedure included polyA-selected RNA extraction, RNA fragmentation, random hexamer primed reverse transcription, and 100nt paired-end sequencing by DNBSEQ-T7. Genes exhibiting a|log2FC|> 1 and an adjusted *P*-value < 0.05. The R package “clusterProfiler” was employed for the enrichment analysis of differentially expressed genes (DEGs). Significant Gene Ontology (GO) and Kyoto Encyclopedia of Genes and Genomes (KEGG) terms were identified as those having a Benjamini-Hochberg (BH) adjusted *P*-value < 0.05.

### Senescence β-galactosidase (SA-β-Gal) activity assay

The Senescence β-galactosidase (SA-β-Gal; Beyotime, China) was utilized to assess SA-β-Gal activity according to the protocol. Senescent chondrocytes were identified as cells exhibiting a blue-stained appearance when observed under light microscopy.

#### Assessment of intracellular ROS

Intracellular ROS were measured using 2′,7′-dichlorodihydrofluorescein diacetate (DCFH-DA) (Beyotime, China), following the manufacturer's instructions. Chondrocytes were initially seeded in six-well plates at a density of 5 × 10^5^ cells per well and pretreated with IGK for 24 h. Next, 400 μM H_2_O_2_ (Sigma, USA) was used to induce ROS in chondrocytes. The chondrocytes were incubated with 10 μM DCFH-DA for 30 min at 37℃. Fluorescence was detected by fluorescence microscopy.

### Statistical analysis

Statistical analyses were performed using GraphPad Prism 9.0. All data are reported as the mean ± SEM. Each experiment was independently replicated at least three times. Normality tests were performed using the Shapiro–Wilk test, and variance homogeneity was assessed with Levene’s test prior to the selection of the appropriate statistical methods. The comparison of multiple samples was determined using one-way ANOVA, and group means were compared using the Student’s t-test. The significance levels were denoted as *, **, ***, and ****, indicating *p* < 0.05, *p* < 0.01, *p* < 0.001, and *p* < 0.0001, respectively. Statistical significance was defined as a *p*-value less than 0.05.

## Result

### Impact of IGK on chondrocyte viability

**Fig. 1:**
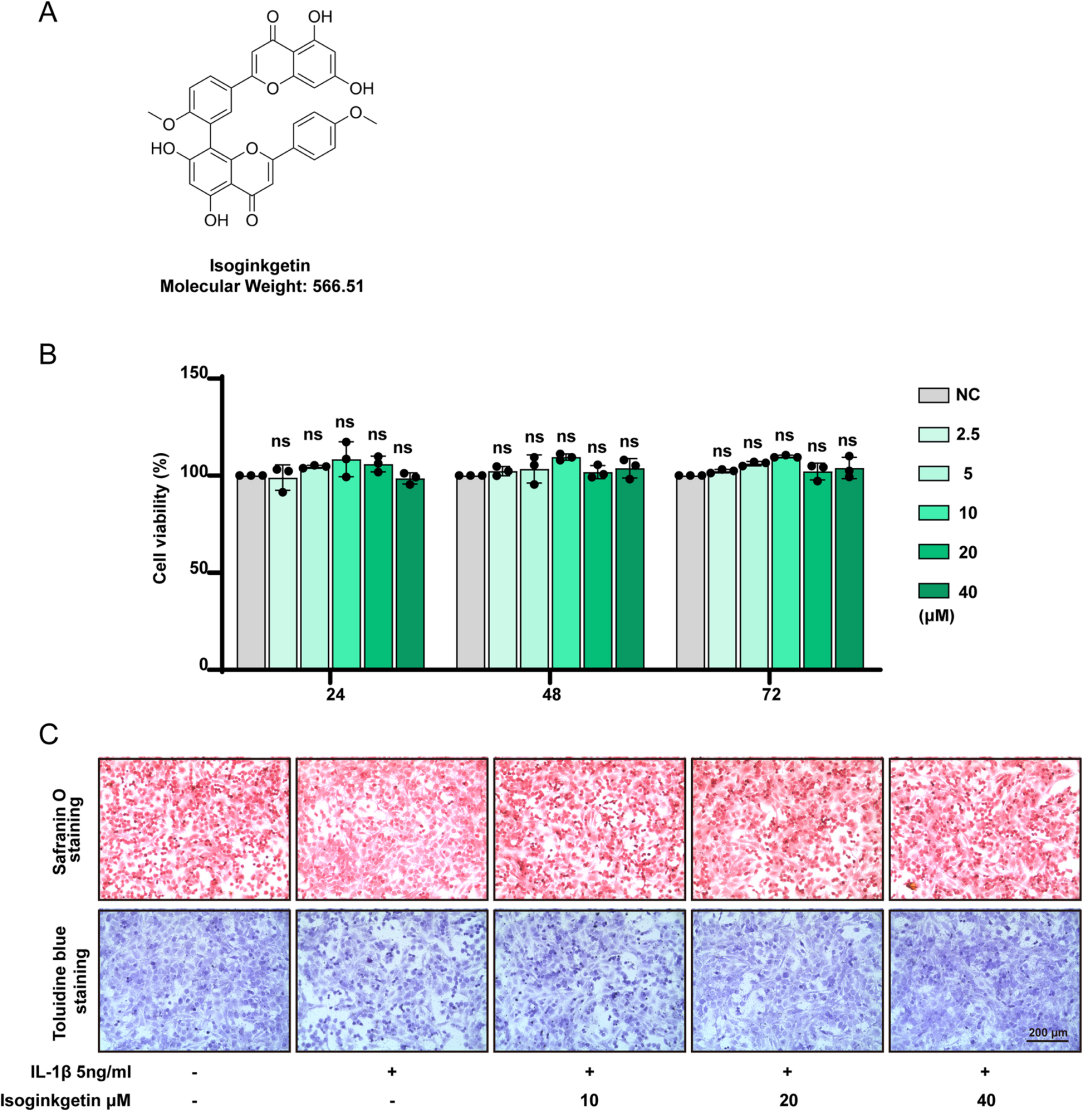
Effect of IGK on the chondrocyte viability. **A** Chemical structure and molecular weight of IGK. **B** The cytotoxic effect of IGK (2.5, 5, 10, 20 and 40 μM) was determined for 24, 48 and 72 h on the chondrocytes. The columns represent the means ± S.D., ns = no significance. **C** Chondrocyte morphology is revealed by toluidine blue staining, scale bar = 200 μm

Figure 1A shows the chemical structure and molecular weight of IGK. The CCK-8 assay was used to evaluate the cytotoxic effects of IGK on chondrocytes. The concentrations of IGK were 2, 4, 6, 8, and 10 μM, and the treatment durations were 24, 48, and 72 h (Fig. 1B). The results indicate that IGK had no significant cytotoxic effects on chondrocytes. The morphology of chondrocytes was examined using toluidine blue and safranin O staining (Fig. 1C). The results showed that chondrocytes treated with IL-1β showed a slight decrease in staining compared to the control group, but this effect was reversed by treatment with IGK.

### IGK reduces cartilage degradation in the DMM model

**Fig. 2:**
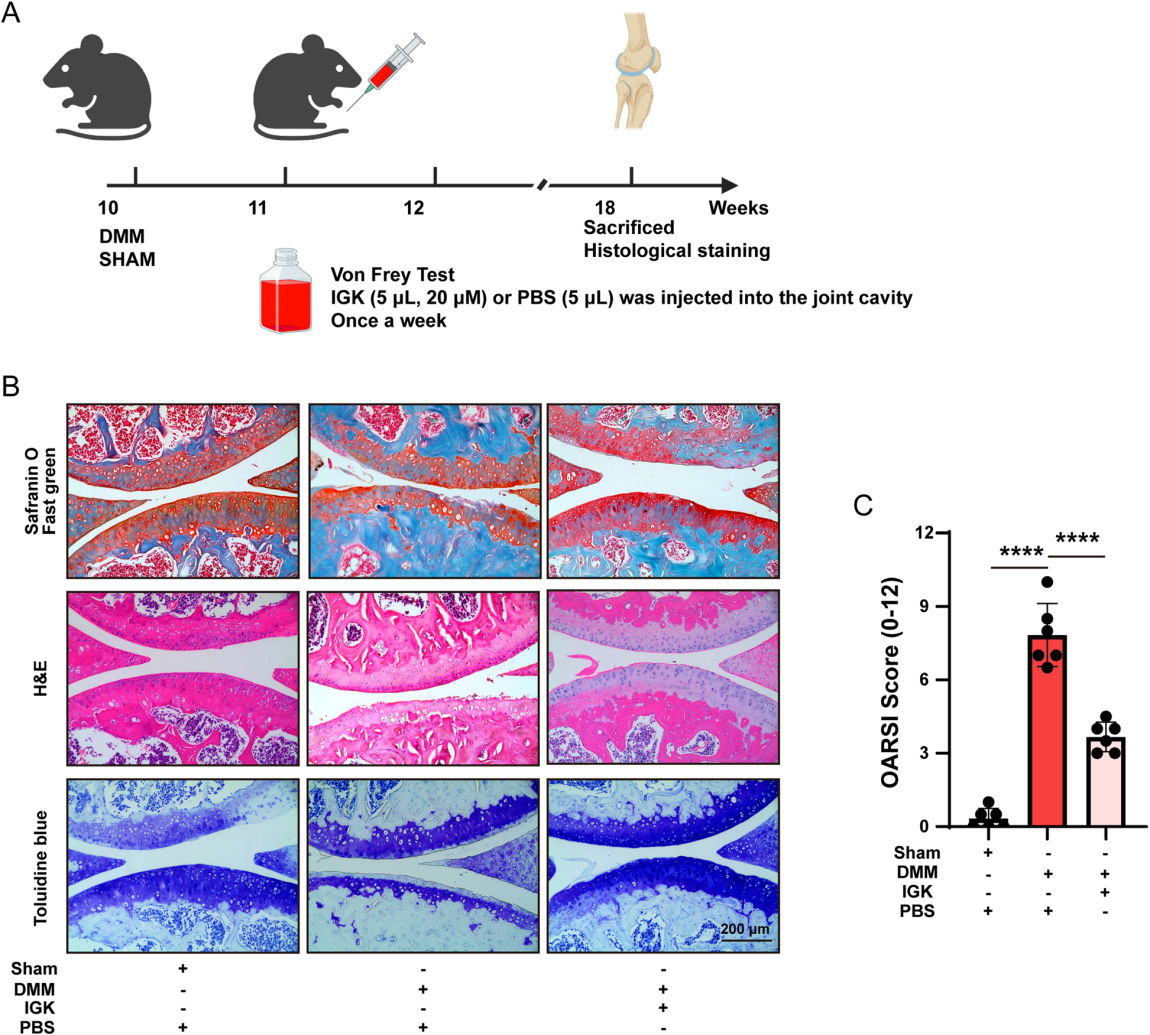
IGK ameliorates cartilage degradation in mice DMM models. **A** Mice flow chart of IGK administration drew by BioRender. **B** Representative images of Safranin O/Fast Green, H&E and Toluidine blue staining in each group, scale bar = 200 μm. **C** Quantitative analysis of the OARSI scores (*n* = 6). Data were presented as means ± SD (*n* = 6). **** *p* < 0.0001

An intra-articular injection of IGK was performed to elucidate how IGK works in DMM models in vivo (Fig. 2A). H&E, toluidine blue and safranin O/fast green staining revealed significant morphological changes (Fig. 2B). The DMM groups showed a disrupted cartilage surface. The red color fading indicates a high loss of proteoglycans in this condition. After 8 weeks of treatment with IGK, the medical professionals identified a smooth cartilage surface. Furthermore, the OARSI score, which measures the extent of articular cartilage damage, confirmed that IGK decelerated the advancement of OA (Fig. 2C). The results suggest that IGK treatment ameliorated the negative effects of articular cartilage degradation caused by DMM models.

### IGK regulates catabolism and anabolism in vitro and in vivo

**Fig. 3:**
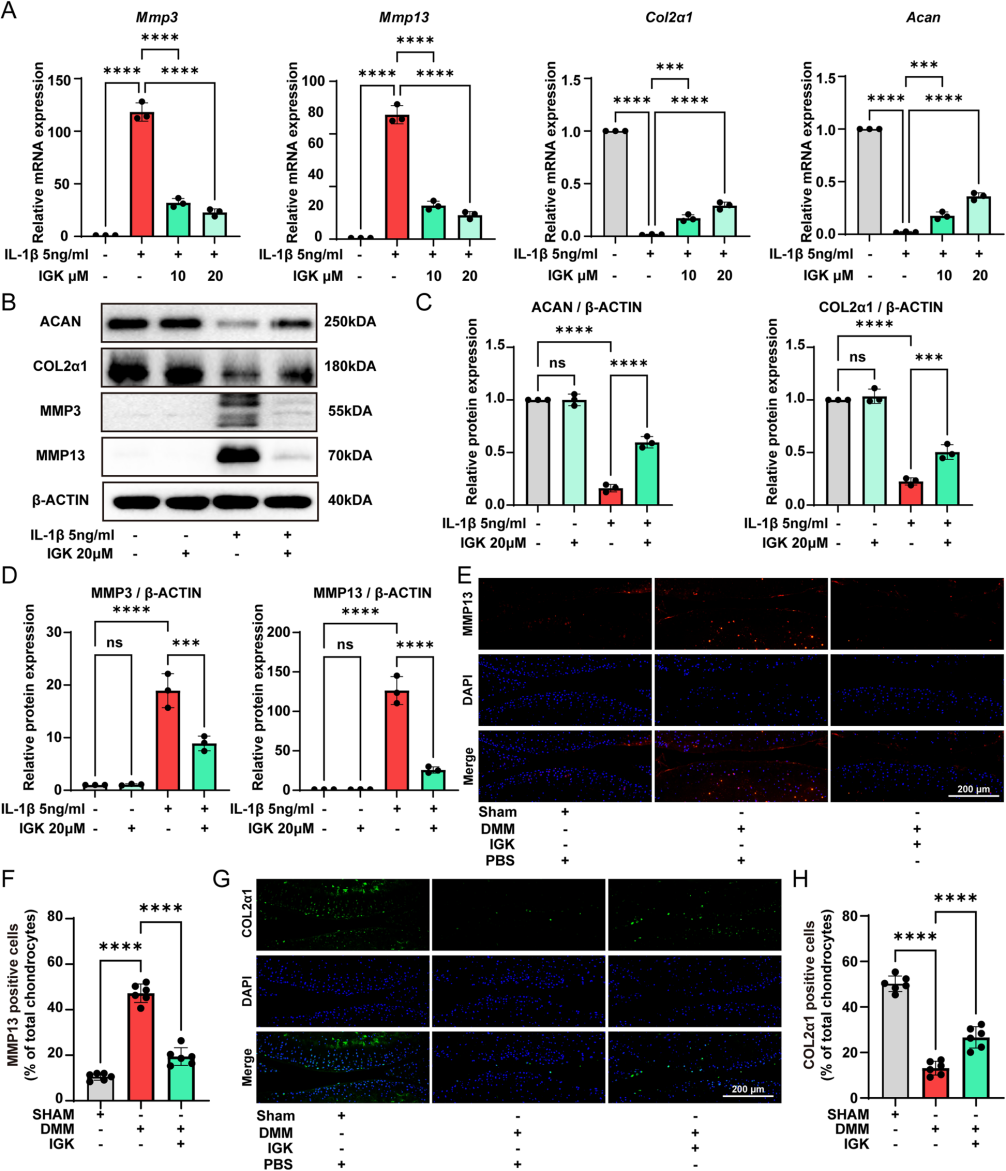
IGK inhibits the catabolism and promotes the anabolism in mice OA chondrocytes in vitro and in *vivo* experiments. **A** The mRNA expression catabolic-related genes (*Mmp3* and *Mmp13*) and anabolic-related genes (*Col2α1* and *Acan*) were measured by RT-qPCR analysis. **B** Western blot analysis and (**C**) quantitative data for anabolic-related proteins (**D**) quantitative data for catabolic-related proteins. **E** Immunofluorescent staining of catabolic protein MMP13 among groups in vivo experiments, scale bar = 200 μm. **F** Quantitative analysis of (**E**). **G** Immunofluorescent staining of catabolic protein COL2α1 among groups in vivo experiments, scale bar = 200 μm. (**H**) Quantitative analysis of (**F**). Data were presented as means ± SD (*n* = 3). ns, no significance; *p < 0.05; **p < 0.01; ***p < 0.001, ****p < 0.0001

Chondrocyte catabolism and anabolism are crucial for maintaining cartilage ECM homeostasis. Any metabolic imbalance contributes to degrading the ECM, leading to the development of OA (Malemud 2004). Treatment of IGK improved the IL-1β-induced decrease in the protein and mRNA levels of anabolism indices, such as ACAN and COL2α1. Conversely, the administration of IGK notably mitigated the elevations in the protein and mRNA levels of catabolism indices, such as MMP3 and MMP13 that were induced by IL-1β (Fig. 3A-D). In vivo, following the induction of DMM, there was a marked up-regulation of MMP13 expression in the knee cartilage of mice. Concurrently, COL2α1 expression was significantly down-regulated. However, after IGK treatment, the levels of MMP13 (Fig. 3E-F) and COL2α1 (Fig. 3G-H) were reversed. The results suggest that IGK suppressed catabolism and promoted anabolism in vitro and in vivo OA models*.*

### IGK reduces pain and inflammation in vivo and in vitro

**Fig. 4:**
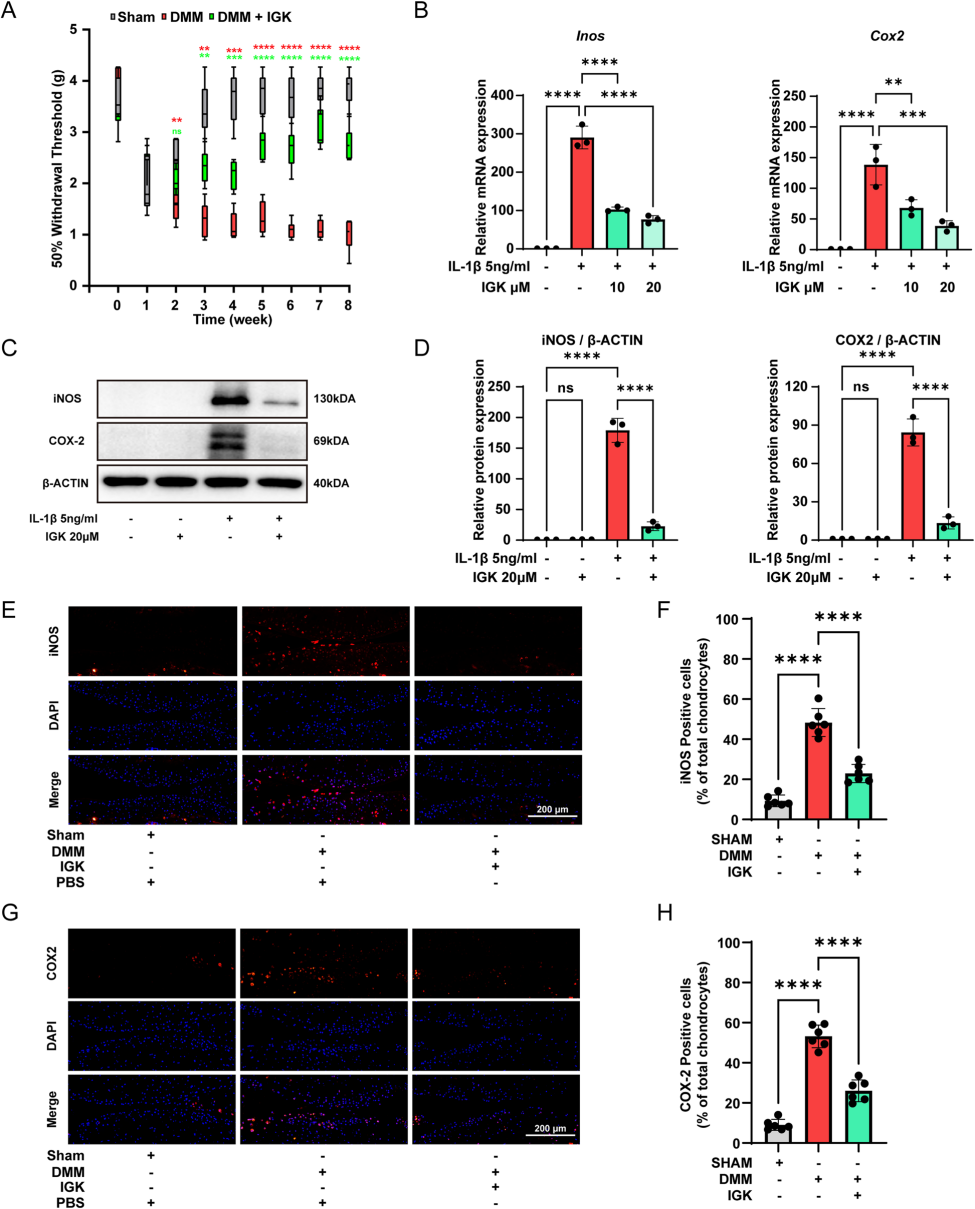
IGK overexpression relieves inflammation and pain in OA. **A** The 50% withdrawal threshold (g) of the right hind paw mechanosensitivity was measured using the von Frey up-down method. **B** The mRNA expression inflammation and pain genes (*iNos* and *Cox-2)* were measured by RT-qPCR analysis. **C** Western blot analysis and (**D**) quantitative data for inflammation and pain proteins (iNOS and COX-2) in chondrocytes. **E** Immunofluorescent staining of iNOS. **F** quantitative analysis of (**E**). **G** Immunofluorescent staining of COX2. **H** Quantitative analysis of (**G**), scale bar = 200 μm. Data were presented as means ± SD (*n* = 3). ns, no significance; *p < 0.05; **p < 0.01; ***p < 0.001, ****p < 0.0001

The main symptom of arthritis is pain. The Von Frey test was used to assess pain sensation in the DMM model of murine OA (Wei et al. 2023). There was no statistically significant difference in pain response prior to DMM surgery. Two weeks after DMM surgery, the mice developed secondary allodynia. Two weeks after IGK administration, the pain in the mice hind paw was alleviated (Fig. 4A). To investigate the anti-inflammatory effect of IGK on OA, chondrocytes were stimulated with IL-1β in vitro to mimic the cellular environment of OA. Inducible nitric oxide synthase (iNOS) promotes the production of nitric oxide (NO). Excess NO is considered a pro-inflammatory mediator that can act as a tissue-damaging molecule and accelerate the progression of OA (Ahmad et al. 2020). Besides, cyclooxygenase-2 (COX-2) is another significant indicator of OA linked to inflammation and pain (Richard et al. 2023). IL-1β increased the mRNA and protein levels of iNOS and COX-2. However, the administration of IGK reversed the inflammatory response enhancement induced by IL-1β (Fig. 4B-D). In vivo, expression of iNOS (Fig. 4E-F) and COX-2 (Fig. 4G-H) was significantly reduced in the DMM + IGK group compared to the DMM group. These results have demonstrated that IGK administration relieves pain and inflammation in OA.

### mRNA sequencing after IGK administration in chondrocytes

**Fig. 5:**
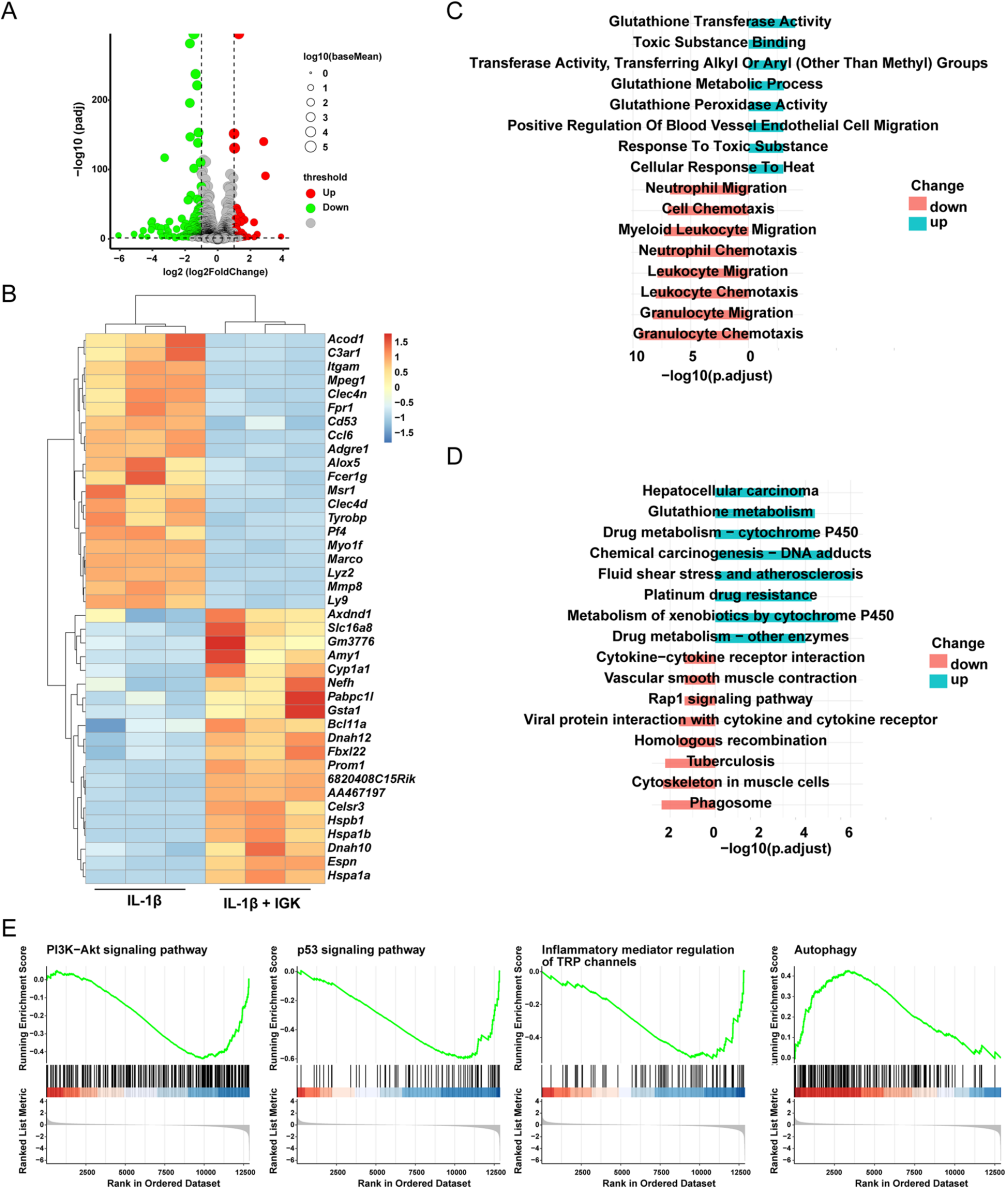
mRNA sequencing after IGK administration in chondrocytes. **A** The volcano plot displays 62 upregulated DEGs and 289 downregulated genes. **B** The heatmap illustrates the TOP 20 up- and down-regulated genes. **C** A bioinformatics analysis of the biological processes represented in the GO database. **D** The KEGG enrichment analysis identified some pathways regulated by IGK. **E** GSEA of DEGs

To explore the mechanism of IGK regulating chondrocyte degeneration, we performed mRNA sequencing in chondrocytes after being stimulated by IL-1β or IL-1β + IGK for 24 h. 62 upregulated and 289 downregulated genes were detected in chondrocytes (Fig. 5A). The top 20 up- and down-regulated genes are represented in Fig. 5B. The GO enrichment analysis (Fig. 5C) and KEGG analysis (Fig. 5D) were performed to show the function and pathway in which the chondrocytes take part after the IGK administration. To further explore the signaling pathway related to OA, GESA analysis was performed (Fig. 5E). The results showed that pathways such as PI3K/AKT, P53, Autophagy, Ferroptosis, Inflammatory mediator regulation of TRP channels pathway were suppressed after IGK administration. These findings have provided evidence suggesting that the modulation of inflammatory and aging-related pathways could potentially participate in the mechanism by which IGK regulates OA.

### IGK inhibits the NF-κB pathways

**Fig. 6:**
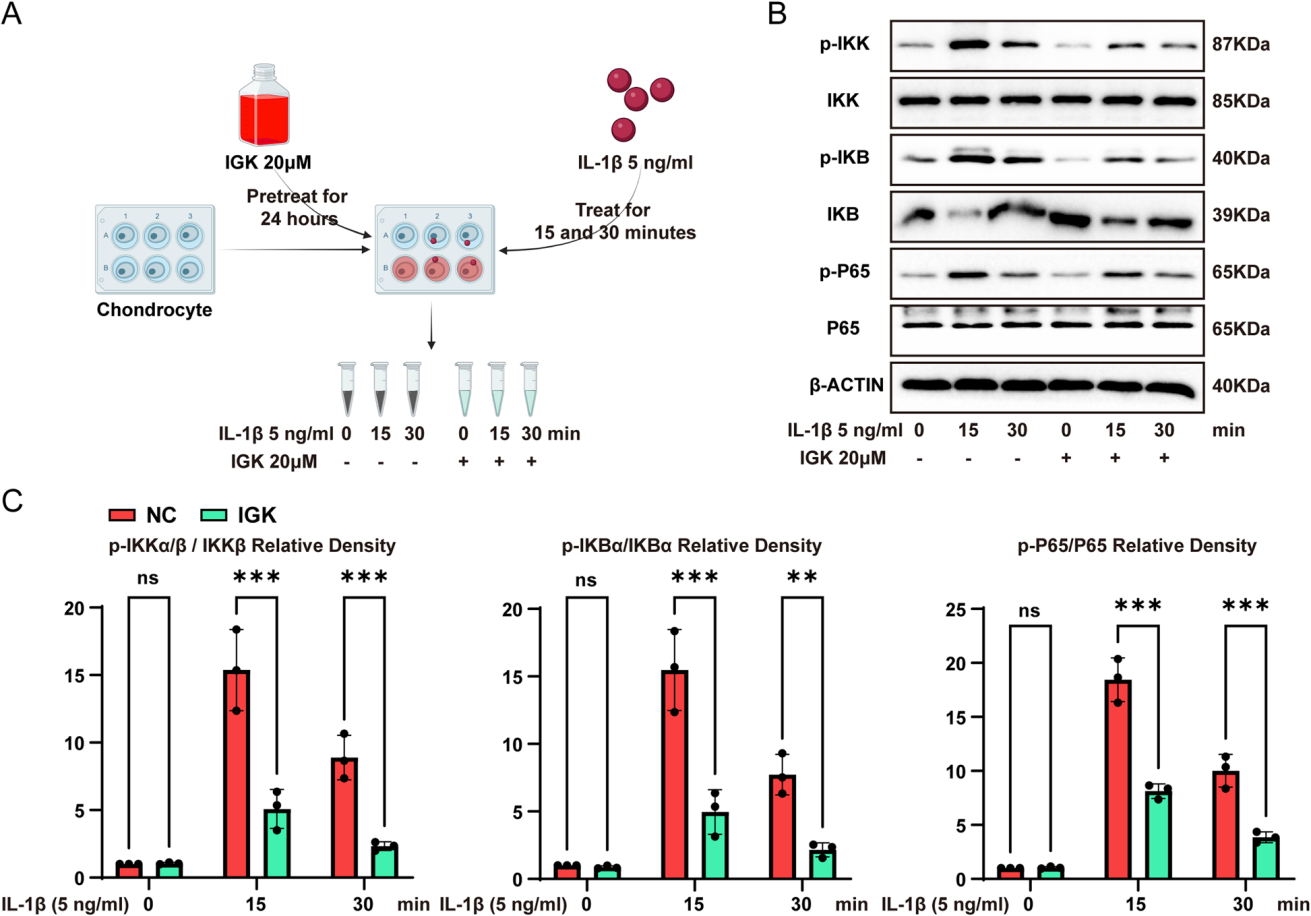
IGK inhibits the NF-κB signalling pathway. **A** Chondrocytes protein used for testing NF-κB signalling pathway flow chart of IGK administration drew by BioRender. **B** Representative western blot of phosphorylated p65, IKKβ, and IκBα after IGK administration in chondrocytes and (**C**) the phosphorylation ratio. Data were presented as means ± SD (*n* = 3). ns, no significance; *p < 0.05; **p < 0.01; ***p < 0.001

As the downstream event of PI3K/AKT signal pathway, the NF-κB signalling pathway, which plays a role in the pathophysiology of OA, is activated by inflammation and ageing (Yao et al. 2023; Li et al. 2019b). We examined the phosphorylation levels of the components of the NF-κB pathway in chondrocytes to determine whether IGK administration affects IL-1β-induced cartilage degradation by modulating the NF-κB signalling pathway (Fig. 6A). After treatment with IGK, the increase in phosphorylation of IKK, IKB and p65 mediated by IL-1β was reduced (Fig. 6B-C). These findings indicate that IGK inhibits the NF-κB signalling pathway.

### IGK alleviates ROS and senescence in chondrocytes

**Fig. 7:**
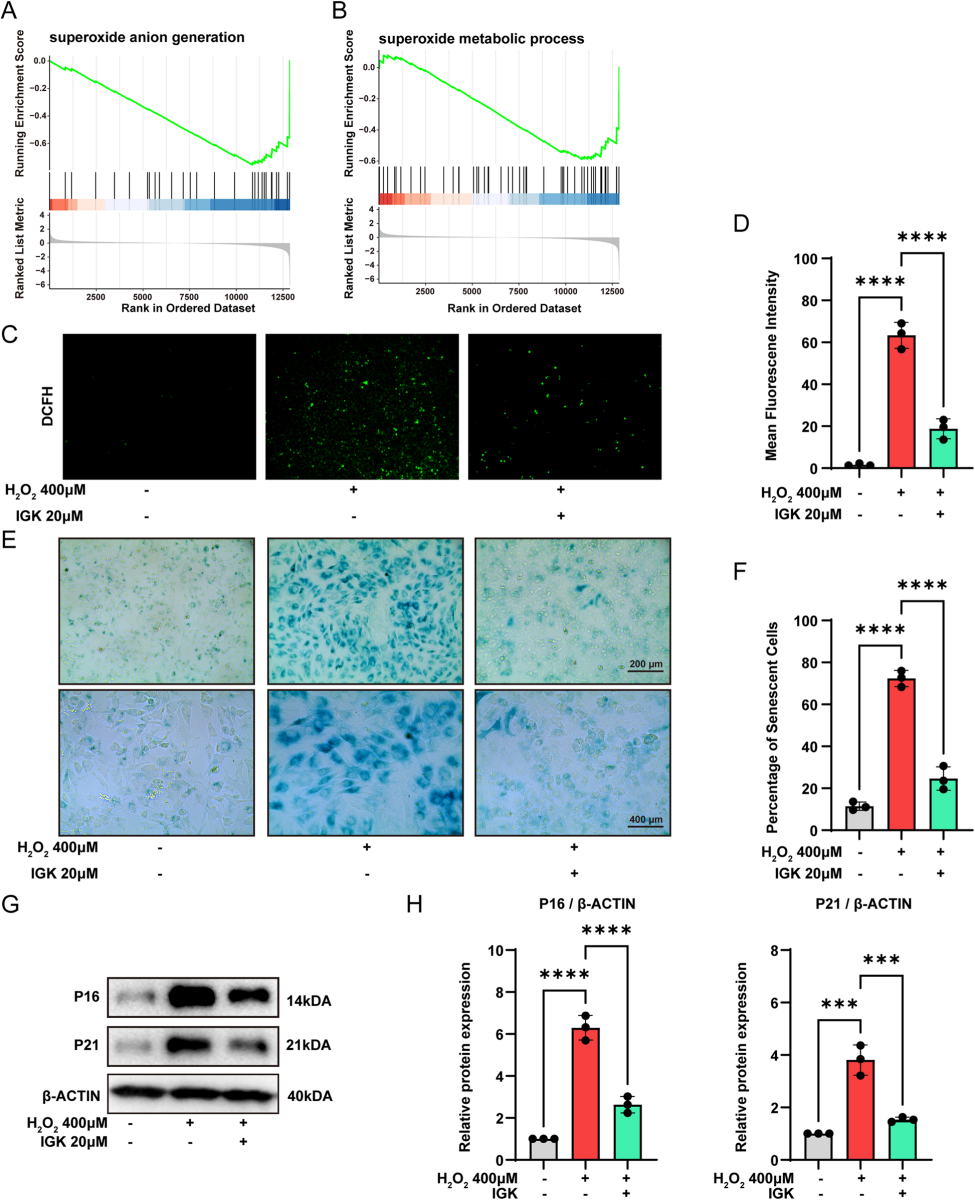
IGK regulates senescence and ROS of chondrocytes. **A** GSEA analysis shows superoxide anion generation. **B** GSEA analysis shows superoxide metabolic process. **C** Representative fluorescence imaging of intracellular ROS and (**D**) mean fluorescence intensity in chondrocytes after IGK administration. **E** SA-β-Gal staining and (**F**) quantification of SA-β-Gal positive rate, scale bar = 200 and 400 μm. **G** Western blot analysis and (**H**) quantitative data for senescence proteins (P16 and P21) in chondrocytes. Data were presented as means ± SD (*n* = 3). ns, no significance; ***p < 0.001, ****p < 0.0001

GSEA analysis showed that Ferroptosis and senescence-related P53 pathways were significantly inhibited after IGK treatment. Ferroptosis is characterized by the abnormal accumulation of ferric ions and ROS induced by lipid peroxidation (Jiang et al. 2021). The accumulation of ROS is implicated in the onset of senescence and the SASP (Davalli et al. 2016; Ren et al. 2023). Besides, through GSEA, we found that two ROS-related pathways, superoxide anion generation and superoxide metabolic process, were inhibited after IGK intervention (Fig. 7A-B). Following H_2_O_2_ stimulation, chondrocytes exhibited a significant increase in oxidative stress, as determined by cell fluorescence. After IGK administration, the ROS level of chondrocytes was decreased (Fig. 7C-D). In addition, we investigated the effects of IGK on the H_2_O_2_-induced senescence model. After H_2_O_2_ treatment, chondrocytes had a higher percentage of SA-β-Gal positive cells (Fig. 7E-F). Furthermore, Western blot analysis revealed increased expression of the senescence biomarkers P16 and P21 in chondrocytes treated with H_2_O_2_ (Fig. 7G-H). The results demonstrate the successful construction of the senescent chondrocytes model. However, IGK partially reversed the changes induced by H_2_O_2_, demonstrating its ability to attenuate senescence and ROS.

## Discussion

OA is a highly prevalent disease that affects multiple joints, including the hip, knee, ankle, hand, and temporal-mandibular joint (Yao et al. 2023). The incidence of OA has increased in recent years as it is a common joint condition. Strategies for treating OA typically include conventional medications, which can have side effects such as liver damage and gastrointestinal discomfort (Marmon et al. 2021). As a result, there has been an increase in research into the pathogenic mechanisms and processes of OA, as well as into potential targets for its treatment.

IGK, a crucial element of traditional Chinese medicine, is derived from *G. biloba*. IGK has been clinically used to treat chest arthralgia, heartache, stroke, and other conditions caused by blood stasis (Tian et al. 2017). Previous studies suggest that IGK enhances Nrf2/ARE signaling, which protects against obesity-induced cardiomyopathy (Wu et al. 2022), attenuates neurotransmitter deficiency and depression-like behaviors through downregulating p38/NF-κB signaling pathway (Li et al. 2021). Besides, IGK ameliorates ECM degradation, apoptosis in nucleus pulposus cells via enhancing autophagy (Yu et al. 2023). IGK and its associated signaling pathways may also play a role in alleviating OA. Previous research has shown that ROS (He et al. 2023), senescence and the SASP (Davalli et al. 2016) have been implicated in cartilage degradation and OA.

The anabolic and catabolic phenotypes of chondrocytes are crucial for the maintenance of extracellular matrix (ECM) homeostasis in cartilage (Singh et al. 2019). Acan and Col2α1 are key indicators of anabolism and are crucial in maintaining the physiological characteristics of cartilage (Lu et al. 2023). Regarding catabolism, MMP3 and MMP13 are the most critical enzymes in chondrocyte catabolism among the many catabolic enzymes. They degrade ACAN and COL2α1, leading to the destruction of ECM (Hu et al. 2021; Wan et al. 2021). In vitro models of OA often use IL-1β as a pro-inflammatory factor. Furthermore, IL-1β promotes catabolic phenotypes (MMP3 and MMP13) and suppresses anabolic phenotypes (ACAN and COL2α1). However, the administration of IGK alleviated this trend. This effect is in line with the anti-degeneration effects of IGK in the intervertebral disc (Yu et al. 2023). Fascinatingly, IGK protected cartilage from destruction and effectively reduced OARSI scores in the DMM-induced OA model. These findings revealed the significant anti-degenerative effects of IGK.

Pain is one of the primary symptoms of OA and a significant contributor to functional decline (Pinals 1996), which is aggravated by iNOS and COX-2 (Dieppe and Lohmander 2005). Von Frey hairs were used to examine evoked, reflexive behaviours (such as paw withdrawal, shake, and lick) at a location distal to the damaged joint (Deuis et al. 2017). Following DMM operation, the 50% withdrawal threshold of the rear right paw was decreased compared to the sham group. However, with IGK administration, the threshold was raised. Besides, in vivo and in vitro, iNOS and COX-2 were suppressed following IGK treatment. Thus, local injection of IGK appears to reduce the neuropathic aspects of OA pain.

Many signaling pathways play a key role in the progression of OA. The PI3K/AKT signalling pathway plays a pivotal role in the pathogenesis of OA, influencing a multitude of processes including synovial inflammation, subchondral bone sclerosis, extracellular matrix (ECM) homeostasis, chondrocyte proliferation, apoptosis, autophagy, and inflammation. These effects significantly impact cell fate and the pathophysiology of OA (Sun et al. 2020). Activation of the PI3K/AKT pathway inhibits autophagy (Xu et al. 2021), while activation of autophagy promotes cell survival at the early stage of OA (Yao et al. 2023). The NF-κB pathway represents a crucial inflammatory mechanism, whose activation is also regulated by the PI3K-AKT pathway (Deng et al. 2024; Liu et al. 2023). The NF-κB pathway plays a pivotal role in the pathogenesis of chondrocyte metabolic dysfunction. NF-κB signaling stimulates the secretion of degrading enzymes like MMP, ADAMTS4, and ADAMTS5, which in turn causes the degradation of articular cartilage (Ulivi et al. 2008). Additionally, many NF-κB regulated cytokines and chemokines (e.g., TNF-α, RANKL, IL-1β, IL-6, and RANK ligands) expressed in OA articular chondrocytes upregulate MMP production, downregulate collagen and proteoglycan synthesis, and enhance NF-κB signaling through a positive feedback loop (Kapoor et al. 2011). Besides, NF-κB can worsen joint injury via inducing PGE2, nitric oxide synthase (NOS), nitric oxide (NO), and COX2. This, in turn, promotes tissue inflammation, catabolic factor synthesis, and apoptosis of articular chondrocytes (Ulivi et al. 2008). In this study, through GSEA analysis, IGK administration inhibited the PI3K pathway and promoted autophagy. Moreover, in the NF-κB pathway, the phosphorylation level of IKKβ, IKBα and P65 was decreased after IGK administration in IL-1β environment. These results suggested that IGK played a protective role in cartilage through PI3K/AKT/NF-κB and autophagy pathway, and regulated the inflammatory status in vitro.

The SASP has been identified in the degradative articular cartilage (Coppé et al. 2011; Tsuchida et al. 2014). Furthermore, the dysfunction of mitochondria related to aging, which leads to oxidative stress, is marked by an excessive buildup of ROS and an imbalance in the energy metabolism of articular chondrocytes (Scott et al. 2010; Blanco et al. 2011). This process is accompanied by p53 and p16, respectively, leading to activation of p21 and cell cycle arrest (Barnes et al. 2019). In addition, inflammation in the joint, which is also related to SASP, leads to destructive changes in the ECM of the articular cartilage and promotes OA (Greene and Loeser 2015). In our research, we found that the p53 pathway was suppressed in IL-1β + IGK group through GSEA analysis. The levels of P21 and P16 were up-regulated in IL-1β group. However, with IGK treatment, the expression levels of P21 and P16 were reduced. These results demonstrated that IGK treatment inhibited chondrocyte senescence and ROS, which could alleviate OA.

In summary, our data showed that IGK reversed the effects of anabolism inhibition, catabolism enhancement, and inflammation promotion in IL-1β induced chondrocytes by suppressing the NF-κB signaling pathway, senescence, and ROS in vitro. In vivo the experiments showed that treatment with IGK could reduce the pain response and attenuate the erosion of knee cartilage and the changes in anabolic and catabolic related markers, such as COL2α1 and MMP13, induced in the DMM group. These findings indicate that IGK will be a potential strategy for OA treatment.

Nevertheless, our research had certain limitations. In our study, we primarily focused on cartilage degradation since it is a central feature of OA. Other OA-related features, such as synovial inflammation and osteophyte formation, are also crucial in OA. These features were not assessed in the present study. In future studies, we will further explore the effects of IGK on synovial inflammation and osteophyte formation. Additionally, the bioavailability and biosafety of IGK need to be explored in future OA treatment.

## Data Availability

We will provide the raw data after the article is received.
